# The Isolation and In Vitro Differentiation of Primary Fetal Baboon Tracheal Epithelial Cells for the Study of SARS-CoV-2 Host-Virus Interactions

**DOI:** 10.3390/v15040862

**Published:** 2023-03-28

**Authors:** Bharathiraja Subramaniyan, Sunam Gurung, Manish Bodas, Andrew R. Moore, Jason L. Larabee, Darlene Reuter, Constantin Georgescu, Jonathan D. Wren, Dean A. Myers, James F. Papin, Matthew S. Walters

**Affiliations:** 1 Department of Medicine, Section of Pulmonary, Critical Care & Sleep Medicine, University of Oklahoma Health Sciences Center, Oklahoma City, OK 73104, USA; Bharathiraja-Subramaniyan@ouhsc.edu (B.S.); Manish-Bodas@ouhsc.edu (M.B.); Andrew-R-Moore@ouhsc.edu (A.R.M.); 2 Department of Obstetrics and Gynecology, University of Oklahoma Health Sciences Center, Oklahoma City, OK 73104, USA; Sunam-Gurung@ouhsc.edu (S.G.); Dean-Myers@ouhsc.edu (D.A.M.); 3 Department of Microbiology and Immunology, University of Oklahoma Health Sciences Center, Oklahoma City, OK 73104, USA; Jason-Larabee@ouhsc.edu; 4 Division of Comparative Medicine, University of Oklahoma Health Sciences Center, Oklahoma City, OK 73104, USA; Darlene-Reuter@ouhsc.edu (D.R.); James-Papin@ouhsc.edu (J.F.P.); 5 Genes & Human Disease Research Program, Oklahoma Medical Research Foundation, Oklahoma City, OK 73104, USA; Constantin-Georgescu@omrf.org (C.G.); Jonathan-Wren@omrf.org (J.D.W.); 6 Department of Pathology, University of Oklahoma Health Sciences Center, Oklahoma City, OK 73104, USA

**Keywords:** COVID-19, SARS-CoV-2, non-human primate, baboon, air–liquid interface, airway epithelium, immune response, innate immunity, inflammation

## Abstract

The mucociliary airway epithelium lines the human airways and is the primary site of host-environmental interactions in the lung. Following virus infection, airway epithelial cells initiate an innate immune response to suppress virus replication. Therefore, defining the virus-host interactions of the mucociliary airway epithelium is critical for understanding the mechanisms that regulate virus infection, including Severe Acute Respiratory Syndrome Coronavirus 2 (SARS-CoV-2). Non-human primates (NHP) are closely related to humans and provide a model to study human disease. However, ethical considerations and high costs can restrict the use of in vivo NHP models. Therefore, there is a need to develop in vitro NHP models of human respiratory virus infection that would allow for rapidly characterizing virus tropism and the suitability of specific NHP species to model human infection. Using the olive baboon (*Papio anubis*), we have developed methodologies for the isolation, in vitro expansion, cryopreservation, and mucociliary differentiation of primary fetal baboon tracheal epithelial cells (FBTECs). Furthermore, we demonstrate that in vitro differentiated FBTECs are permissive to SARS-CoV-2 infection and produce a potent host innate-immune response. In summary, we have developed an in vitro NHP model that provides a platform for the study of SARS-CoV-2 infection and other human respiratory viruses.

## 1. Introduction

The pseudostratified mucociliary airway epithelium is a continuous, multicellular tissue that lines the proximal (nasal cavity, trachea and bronchi) and distal (i.e., non-cartilaginous bronchioles) airways of the human respiratory tree [1,2,3,4,5,6,7,8,9]. The primary function of the mucociliary epithelium is to provide a first line of defense and physical barrier that protects the lung from inhaled environmental insults, including particulates and respiratory pathogens (e.g., viruses) [1,2,3,4,5,6,7,8,9]. Luminal facing secretory and ciliated cells are the major cell populations of the mucociliary epithelium and play a critical role in maintaining efficient barrier function via the process of mucociliary clearance [1,2,3,4,5,6,7,8,9]. Despite their critical role in maintaining efficient barrier function, the presence of secretory and ciliated cells at the luminal surface makes them ideal targets for primary infection by multiple respiratory viruses, including influenza A virus (IAV), rhinovirus, respiratory syncytial virus (RSV) and Severe Acute Respiratory Syndrome Coronavirus 2 (SARS-CoV-2), the causative agent of the coronavirus disease 2019 (COVID-19) global pandemic [10,11,12,13,14,15]. Virus binding to its host receptor on the surface of target cells leads to virus entry and release of its genome into the cell to initiate virus replication [10,11,12,13,14,15]. In response to respiratory virus infection, airway epithelial cells initiate a robust innate immune response that leads to the induction of proinflammatory cytokines and chemokines and anti-viral interferons (IFNs) to help suppress virus replication and limit the spread of infection [10,11,12,13,14,15]. Therefore, defining the virus-host interactions at the mucociliary airway epithelial cell interface is critical for understanding the mechanisms that regulate respiratory virus infection and identifying potential targets for the development of novel antiviral therapeutic strategies.

The unexpected and rapid emergence of the COVID-19 global pandemic was accompanied by a high level of morbidity and mortality, which placed an enormous strain on society and health care systems [16,17,18]. To counter this global emergency, the scientific community responded in an unprecedented manner by developing both in vitro and in vivo animal models of SARS-CoV-2 infection to better understand the mechanisms that regulate virus tropism, transmission and pathogenesis [19,20,21,22,23,24,25,26,27,28,29,30,31,32,33,34]. Furthermore, these same models were utilized to develop novel therapeutic agents and vaccines to reduce COVID-19 morbidity and mortality [19,20,21,22,23,24,25,26,27,28,29,30,31,32,33,34]. Based on their anatomical, physiological and immunological similarities to humans, non-human primates (NHP) provide a highly relevant and reliable model for the study of human respiratory disease [35,36]. Studies with rhesus macaques (*Macaca mulatta*), African green monkeys (*Chlorocebus aethiops*), cynomolgus macaques (*Macaca fascicularis*), common marmosets (*Callithrix jacchus*) and baboons (*Papio hamadryas*) have proved pivotal in furthering our understanding of SARS-CoV-2 pathogenesis and the development of COVID-19 therapeutics and vaccine-based countermeasures [37,38,39,40,41,42,43]. However, despite their usefulness for the study of SARS-CoV-2 and other human respiratory virus infections, in vivo NHP models require important ethical considerations for their use in biomedical research, are prohibitively expensive and sometimes fail to fully recapitulate the full aspects and severity of human disease [26,37,39,40,41,44]. Therefore, there is a need to develop physiologically relevant in vitro NHP models of human respiratory virus infection that would allow for rapidly characterizing the suitability of a specific NHP to model the human infection before embarking on in vivo studies.

To address this need, we have developed methodologies for the isolation, in vitro expansion, cryopreservation, and differentiation of primary fetal baboon tracheal epithelial cells (FBTECs) from the olive baboon (*Papio anubis*). Our results demonstrate that FBTECs collected by brush biopsy of the trachea from baboon fetuses delivered by Cesarean section are capable of expansion in vitro and stain positive for the basal cell (BC) marker KRT5. Similar to human BCs, FBTECs are capable of differentiating on air-liquid interface (ALI) culture to form a pseudostratified mucociliary epithelium with secretory and ciliated cells. Importantly, the ALI-differentiated FBTEC cultures express the SARS-CoV-2 entry receptor angiotensin converting enzyme II (ACE2) and entry factor transmembrane protease serine 2 (TMPRSS2) and are permissive to infection with multiple SARS-CoV-2 variants (Washington, Beta, Delta and Omicron). Furthermore, additional studies with the Omicron variant revealed that, similar to infection of human airway epithelial cells [12,15,45,46,47,48,49], SARS-CoV-2 infection of FBTECs resulted in the production of infectious virus and a potent host innate-immune response. In summary, we have developed an in vitro NHP model of the mucociliary epithelium using primary FBTECs that provides a platform for the study of SARS-CoV-2 infection and potentially other human respiratory viruses.

## 2. Materials and Methods

### 2.1. Animals

The experiments utilizing olive baboons (*Papio anubis*) were performed in compliance with guidelines established by the Animal Welfare Act for housing and care of laboratory animals and according to the guidelines in the protocol (Protocol Number: 22-025-AHU), which was approved by the Institutional Animal Care and Use Committee (IACUC) of the University of Oklahoma Health Sciences Center (OUHSC). Throughout the course of the study, the animals were fed twice daily, as well as receiving a daily supplement of fruits. A total of *n* = 8, adult, pregnant female olive baboons were available for sampling the fetal tissue in this study, as they were already scheduled for the outlined procedures for a separate study. At late gestation (168 ± 1 days [0.9 gestation], normal term ~183 days), the animals were sedated with ketamine, maintained on isoflurane, and a Cesarean section was performed to deliver the fetus. Following delivery by cesarean section, euthanasia solution was administered via the umbilical vein to the fetus. Fetal airway epithelial cells were quickly sampled by brush biopsy of the trachea using a nylon bristle cytology brush (catalog number 25–2188, Puritan Medical Products, Guilford, ME, USA) and the cells detached from the brush by flicking into ice cold BronchiaLife epithelial airway medium (BLEAM) (catalog number LL-0023; Lifeline Cell Technology, Frederick, MD, USA) supplemented with penicillin (100 U/mL)–streptomycin (100 μg/mL) (+Pen/Strep) (catalog number 15140122, Thermo Fisher Scientific, Waltham, MA, USA) and stored on ice until further processing as described below. In addition to the brush biopsy, a sample of whole tracheal tissue was collected in 4% paraformaldehyde (catalog number P6148, Sigma Aldrich, St. Louis, MO, USA) and fixed for 48 h at 4 °C. Following fixation, the tracheal tissue was transferred to 70% ethanol and then paraffin embedded using the standard protocol.

### 2.2. Isolation, Expansion and Cryopreservation of Fetal Baboon Tracheal Epithelial Cells (FBTECs)

Airway epithelial cells collected by brushing the fetal trachea (*n* = 5 males and *n* = 3 females) were pelleted by centrifugation (250× *g*, 5 min at room temperature) and disaggregated by resuspension in 5 mL of 0.05% trypsin-ethylenediaminetetraacetic acid (EDTA) (catalog number 25300062, Thermo Fisher Scientific) for 5 min at 37 °C. Trypsinization was stopped by the addition of 15 mL of HEPES buffered saline (catalog number CC-5024, Lonza, Morristown, NJ, USA) supplemented with 15% fetal bovine serum (FBS) (catalog number 10082147, Thermo Fisher Scientific). Following mixing of the sample by gently pipetting up and down ten times, the cells were pelleted by centrifugation (250× *g*, 5 min at room temperature). The cell pellet was then resuspended in 20 mL of room temperature 1X PBS and centrifuged (250× *g*, 5 min at room temperature). Following centrifugation, the cell pellet was resuspended in 5 mL of BLEAM + Pen/Strep, seeded into a T-25 cm^2^ flask (catalog number 430639, Corning^®^, Corning, NY, USA) pre-coated with human type IV collagen (catalog number C7521, Sigma Aldrich) and maintained in a humidified atmosphere with 5% CO_2_ at 37 °C (standard conditions for all cell culture experiments performed in this study). The following day, the unattached cells were removed by changing the media with fresh BLEAM + Pen/Strep, and thereafter, the media was changed every 2–3 days until the cells had reached 70–80% confluence in the flask (defined as Passage 0 cells). Once the Passage 0 cells had reached the desired confluence, they were harvested by trypsinization and then neutralized as described above. The cells were then resuspended in 15 mL of BLEAM + Pen/Strep and seeded into an uncoated T-75 cm^2^ flask (catalog number 430641U, Corning^®^). The next day, the media was replaced on the cells, with additional media changes every 2–3 days until the cells reached 70–80% confluence (defined as Passage 1 cells). The Passage 1 cells were then harvested by trypsinization, neutralized, and cryopreserved via resuspension in freezing media (density of >1.5 × 10^5^ cells per 1 mL) consisting of BLEAM + Pen/Strep supplemented with 10% FBS and 10% DMSO (catalog number D26501, Sigma Aldrich). The resuspended cells were then incubated for 5 days at −80 °C inside a Corning^®^ CoolCell^®^ LX freezing container (catalog number 432138, Corning^®^), before being transferred to liquid nitrogen for long term storage.

### 2.3. Air-Liquid Interface (ALI) Culture

Cryopreserved Passage 1 FBTECs were thawed rapidly and resuspended in a total volume of 15 mL of BLEAM + Pen/Strep. The cells were then seeded into an uncoated T-75 cm^2^ flask (cell density > 3000/cm^2^) and cultured in the same manner as described above, with fresh media replaced the day after seeding. Once the cells had reached 70–80% confluence, they were harvested via trypsinization and differentiated into a pseudostratified mucociliary airway epithelium on ALI culture. Briefly, 1 × 10^5^ FBTECs in 100 μL of BLEAM + Pen/Strep media were seeded in the apical chamber of a Transwell^®^ insert (catalog number 3470, Corning^®^) pre-coated with human type IV collagen, with 1 mL of BLEAM + Pen/Strep added to the basolateral chamber (ALI day-2). The next day (ALI day-1), fresh BLEAM + Pen/Strep media was replaced in both the apical (100 μL) and basolateral (1 mL) chambers. Twenty-four hours later (ALI day 0), the media was removed from the apical chamber to expose the cells to air, and 1 mL of HBTEC ALI differentiation medium (catalog number LM-0050, Lifeline^®^ Cell Technology) + Pen/Strep was added to the basolateral chamber. The apical chamber was exposed to air for the remainder of the experiment, with media in the basolateral chamber replaced every 2–3 days.

### 2.4. Immunofluorescence Staining

In vitro FBTEC cultures (pre and post ALI), paraffin-embedded sections of ALI wells or paraffin-embedded in vivo baboon fetus trachea, were stained as previously described [50]. Primary antibodies against KRT5 (2 µg/mL, catalog number PA1-37974, Thermo Fisher Scientific), SCGB1A1 (5 µg/mL, catalog number RD181022220-01, BioVendor LLC, Asheville, NC, USA), acetylated tubulin (5 µg/mL, catalog number T7451, Sigma Aldrich), KRT5 (2 µg/mL, catalog number MAB1620, Sigma Aldrich), and SARS-CoV-2 nucleocapsid (10 µg/mL, catalog number MA1-7403, Thermo Fisher Scientific) were used in conjunction with the secondary antibodies, goat anti-mouse Alexa Fluor 488 (2 µg/mL, catalog number A11029, Thermo Fisher Scientific) and goat anti-rabbit Alexa Fluor 546 (2 µg/mL, catalog number A11035, Thermo Fisher Scientific). For each staining, the cell nuclei were counterstained with DAPI (1 µg/mL, catalog number 62248, Thermo Fisher Scientific). To confirm the specificity of each primary antibody on the in vitro FBTEC cultures or in vivo baboon tissue, staining of comparable human samples was performed in parallel as positive controls. For quantification of the SCGB1A1 and acetylated tubulin-positive cells, *n* = 10 random images were taken, and a minimum of 6000 cells were counted using ImageJ software (version 1.8.0_112, NIH) and normalized to the number of nuclei.

### 2.5. Transepithelial Electrical Resistance (TEER)

The TEER of ALI day 28 cultures was measured using the ENDOHM-6G and EVOM2 apparatus (World Precision Instruments, Sarasota, FL, USA) according to the manufacturer’s guidelines. The resistance (ohms) of an empty Transwell^®^ insert (with no cells) was subtracted from each sample to calculate the true tissue resistance, which was subsequently multiplied by the area of the Transwell^®^ insert (0.33 cm^2^). For each donor, the TEER was measured in *n* = 4 ALI wells, and the mean was used as the final value.

### 2.6. Bulk RNA Sequencing (Bulk RNA-Seq)

On ALI day 28 differentiated FBTECs (*n* = 8 donors) were harvested and the genome-wide transcriptome assessed by bulk RNA-Seq. Libraries were prepared from total RNA for each sample using the QuantSeq 3′ mRNA-Seq Library Prep Kit FWD from Illumina (Lexogen, Vienna, Austria), and the sequencing of each library was performed on a NextSeq 2000 P2 Flowcell (Illumina, San Diego, CA, USA). The raw sequencing reads (in a FASTQ format) were trimmed of the residual adaptor sequences using Scythe software, and the low-quality bases at the beginning or end of the sequencing reads were removed using sickle. The quality of the remaining reads was confirmed with the FastQC utility. Trimmed quality reads were aligned to the Olive baboon (*Papio anubis*) genome Panu_3.0 (Ensembl release 104) using STAR v2.4.0h [51]. Gene-level read counts were determined using featureCounts/Subread v2.0.4 with Papio_anubis. Panu_3.0.104.gtf Ensembl annotations [52]. Read-count normalization and differential expression analyses were performed using the edgeR package from Bioconductor, following the limma/voom workflow [53]. The raw data from the bulk RNA-Seq studies are publicly available at the Gene Expression Omnibus (GEO) site (http://www.ncbi.nlm.nih.gov/geo/, accessed on 7 March 2023), accession number GSE226820.

### 2.7. Western Blotting

Differentiated ALI day 28 samples were harvested and processed for Western blotting analysis as previously described [50]. The following primary antibodies were used: ACE2 (1:1000, catalog number NBP2-67692, Novus Biologicals, Centennial, CO, USA), TMPRSS2 (1:1000 dilution, catalog number NBP238263, R&D Systems, Minneapolis, MN, USA) and GAPDH (1:5000 dilution, catalog number 2118S, Cell Signaling Technologies, Danvers, MA, USA). See Supplementary Figures S1 and S2 for the original Western blot images. Cell lysates of the human airway epithelial cell line BCi-NS1.1 over-expressing human ACE2 (BCi-ACE2) were used as a positive control to confirm the specificity of the human antibodies. To generate this cell line, BCi-NS1.1 cells were infected with a replication-deficient lentivirus expressing untagged human ACE2 under the control of the eukaryotic translation elongation factor 1 α (EF-1α) promoter at a multiplicity of infection (MOI) of 0.1 as previously described [54]. At 2 days post-infection, the cells were treated with 500 µg/mL of G418/Neomycin (catalog number 30-234-CR, Corning^®^) for 2 weeks to select for transduced cells and generate a stable cell line constitutively over-expressing human ACE2 (BCi-ACE2). The BCi-ACE2 cells were subsequently cultured in an identical manner to the parental BCi-NS1.1 cells using BLEAM + Pen/Strep in a humidified atmosphere with 5% CO_2_ at 37 °C. The ACE2-expressing lentivirus (catalog number EX-U1285-Lv160) and its empty vector control (catalog number EX-NEG-Lv160) were purchased from GeneCopoeia, Inc. (Rockville, MD, USA) and generated as previously described [50].

### 2.8. Generation and Titration of Severe Acute Respiratory Syndrome-Related Coronavirus 2 (SARS-CoV-2) Stocks

The following SARS-CoV-2 isolates were obtained through BEI Resources, NIAID, NIH (Manassas, VA, USA): Washington (isolate USA-WA1/2020, catalog number NR-52281), Beta (isolate hCoV-19/USA/MD-HP01542/2021, Lineage B.1.351, catalog number NR-55282), Delta (isolate hCoV-19/USA/MD-HP05647/2021, Lineage B.1.617.2 Delta variant, catalog number NR-55672) and Omicron (isolate hCoV-19/USA/MD-HP20874/2021, Lineage B.1.1.529, Omicron variant, catalog number NR-56461). Viral stocks of Washington, Beta and Delta were produced in Vero E6 cells, whereas Omicron was produced in Calu-3 cells, as previously described [55]. Virus stocks were titrated using the 50% tissue culture infectious dose (TCID_50_) method as previously described [55]. All the experiments involving SARS-CoV-2 were performed in the High Containment Biosafety Level-3 Laboratory Core at OUHSC, according to the guidelines in the protocol (Protocol Number: 100492) approved by the Institutional Biosafety Committee (IBC).

### 2.9. Infection of ALI Cultures

Differentiated ALI cultures were infected with SARS-CoV-2 and harvested for analysis as previously described [55]. Briefly, on the day of infection, the media in the basolateral chamber was replaced with 1 mL of fresh HBTEC ALI differentiation medium + Pen/Strep. The ALI cultures were then infected with SARS-CoV-2 by adding 100 μL of inoculum to the apical chamber of the Transwell^®^ insert at a MOI of 0.05 (average of 1.71 × 10^4^ PFU per ALI well) or 1 (average of 3.14 × 10^5^ PFU per ALI well), calculated based on the total number of cells per ALI well at the time of infection. At each time point post-infection (24–72 h), mock- or SARS-CoV-2-infected cells were collected for RNA extraction or quantification of virus production by TCID_50_ assay using Vero E6-TMPRSS2-T2A-ACE2 cells (catalog number NR-54970, BEI Resources), as described above. In addition, the medium from the basolateral chamber of the mock- or SARS-CoV-2-infected cells was collected and stored at −80 °C.

### 2.10. RNA Extraction, cDNA Synthesis and qPCR Analysis

RNA extractions, cDNA synthesis and quantitative PCR (qPCR) analysis of the SARS-CoV-2 nucleocapsid gene expression were performed as previously described [55]. For qPCR, all samples were analyzed in duplicate, with relative expression levels determined using the dCt method with baboon actin beta (ACTB) as the endogenous control. Expression of baboon ACTB (For primer 5′-GGGAAATCGTGCGTGACATT-3′ and Rev primer 5′-AGGTAGTTTCGTGGATGCCA-3′), IL-1β (For primer 5′-TGAAAGCTCTCCACCTCCAG-3′ and Rev primer 5′-TTGGGCAGACTCGAATTCCA-3′), IL-6 (For primer 5′-ATGCAATAACCACCCCTGAA -3′ and Rev primer 5′-CTGCAGCCACTGGTTCTGT-3′) and CXCL8 (For primer 5′-CCTTTCCACCCCAAA TTTATC-3′ and Rev primer 5′-TTCTGTATTGACGCAGTGTGG-3′) were quantified using the following cycling parameters: 95 °C for 1.5 min, 40 cycles of 95 °C for 45 s, 60 °C for 30 s and 72 °C for 45 s, followed by 95 °C for 1 min and a melt curve of 55 °C to 95 °C (with 0.5 °C increments) for 10 s. Whereas, the expression of baboon IFNL1 (For primer 5′-CGCCTTGGAAGAGTCACTCA-3′ and Rev primer 5′-GAAGCCTTAGGTCCCAATTC-3′) and IFNL3 (For primer 5′-ACATAGCCCAGTTCAAGTC-3′ and Rev primer 5′-GACTCTT CTAAGGCATCTTTG-3′) were quantified with the following cycling parameters: 95 °C for 1.5 min, 40 cycles of 95 °C for 45 s, 55 °C for 30 s and 72 °C for 45 s, followed by 95 °C for 1 min and a melt curve of 55 °C to 95 °C (with 0.5 °C increments) for 10 s. The final working concentration of each primer was fixed at 2.5 ng/µL. For each time point and condition, the gene expression levels were assessed in *n* = 3 ALI wells.

### 2.11. Cytokine, Chemokine and IFN Analysis

A custom MILLIPLEX^®^ Non-Human Primate Cytokine/Chemokine/Growth Factor Panel (catalog number PRCYTA-40K, MilliporeSigma, Burlington, MA, USA) was used to quantify the protein levels of *n* = 9 cytokines, chemokines and IFN signaling-associated proteins (CCL2, CCL3, CCL5, CCL20, CSF2, CXCL8, CXCL10, IFNL2, and IL-6) from culture media supernatants obtained from SARS-CoV-2-infected cultures and uninfected controls. The assays were performed according to the manufacturer’s instructions, using 50 µL of undiluted culture media for each sample in duplicate. The processed samples were subsequently read and analyzed using a Bio-Plex 200 suspension array system (Bio-Rad, Hercules, CA, USA). For each time point and condition, the protein levels were assessed in the medium from the basolateral chamber of *n* = 1 ALI well.

### 2.12. Statistics

A Mann–Whitney U test was used to compare changes between SARS-CoV-2-infected cultures vs. uninfected controls, with a *p*-value of ≤0.05 considered a significant change. All statistical analysis was performed using IBM SPSS Statistics for Windows, Version 27.0 (IBM Corp., Armonk, NY, USA).

## 3. Results

### 3.1. In Vitro Expanded FBTECs Differentiate on ALI Culture to form a Pseudostratified Mucociliary Epithelium

**Figure 1:**
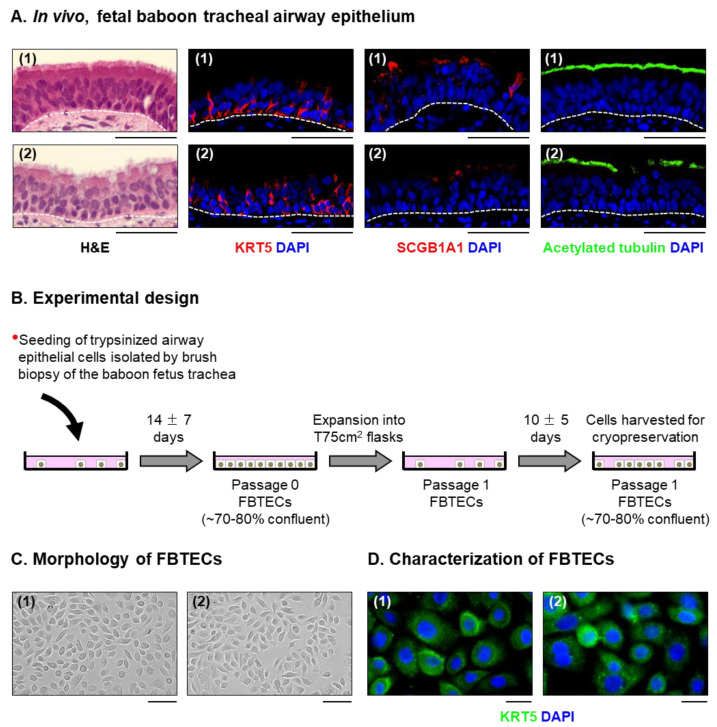
Isolation, in vitro expansion and cryopreservation of primary FBTECs. (**A**) Histological analysis of the in vivo fetal baboon tracheal mucociliary airway epithelium. H&E staining and immunofluorescent staining of BCs (KRT5, red), club cells (SCGB1A1, red) and ciliated cells (acetylated tubulin, green). The nuclei are stained blue with DAPI. Scale bar = 50 µm. Representative images from *n* = 2 baboon fetuses (1–2) are shown. (**B**) Experimental design. Schematic for the isolation, in vitro expansion and cryopreservation of FBTECs. (**C**) Morphology of cultured FBTECs. Scale bar = 50 µm. (**D**) Characterization of FBTECs. Immunofluorescent staining of the BC marker, KRT5 (green), in Passage 1 FBTECs. Nuclei are stained blue with DAPI. Scale bar = 50 µm. Representative images from *n* = 2 FBTEC donors (1–2) are shown in panels (**C**,**D**).

The human pseudostratified mucociliary airway epithelium consists of several cell types, with BC, secretory (mucus producing ‘goblet’ cells or non-mucus producing ‘club’ cells) and ciliated cells encompassing the four major cell populations [1,2,3,4,5,6,7,8,9]. To determine the presence of these cell types in late stage gestation baboon fetuses, we performed immunofluorescent staining of in vivo fetal baboon trachea using cell-type specific markers. Similar to human trachea [1,2,3,4,5,6,7,8,9], our staining demonstrated the presence of KRT5 + BCs, SCGB1A1+ club cells and acetylated tubulin+ ciliated cells (Figure 1A). Despite the confirmation of positive staining in human tissue, we were unable to detect the presence of MUC5AC+ goblet cells in the fetal baboon trachea. BCs are the resident stem/progenitor cells of the human mucociliary airway epithelium that are capable of differentiating into secretory and ciliated cells during normal homeostatic turnover of the epithelium or during repair and regeneration of the epithelium following injury [4,56,57]. Based on their capacity to retain their proliferative and stem/progenitor capacity ex vivo, human BC can be isolated from in vivo airway epithelial samples and expanded in vitro for subsequent cryopreservation [58,59,60]. Therefore, we applied this approach to isolate FBTECs from airway epithelial cells collected by brushing the in vivo fetal trachea (Figure 1B). The in vitro expanded FBTECs displayed a healthy cobblestone morphology typical for human BCs (Figure 1C) and stained positive for the BC marker KRT5+ (Figure 1D).

**Figure 2:**
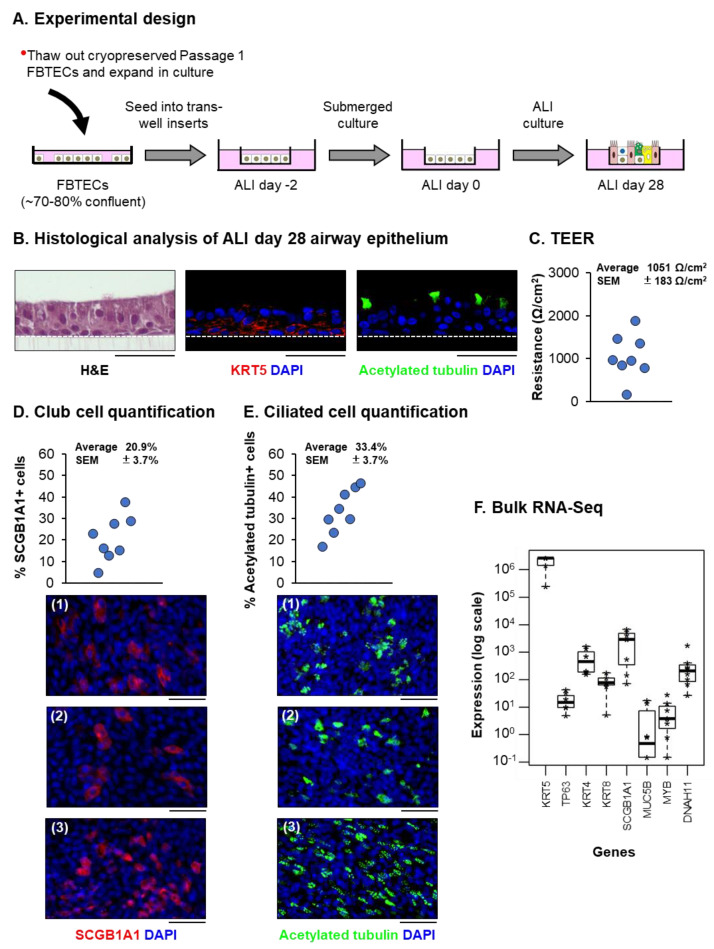
FBTECs differentiate on in vitro ALI cultures to form a pseudostratified mucociliary airway epithelium. (**A**) Experimental design. Schematic for the in vitro differentiation of FBTECs on ALI culture. (**B**) Histological analysis of ALI day 28 airway epithelium. H&E staining and immunofluorescent staining of BCs (KRT5, red) and ciliated cells (acetylated tubulin, green). The nuclei are stained blue with DAPI. Scale bar = 50 µm. Representative images from a single FBTEC donor are shown. (**C**) TEER of ALI day 28 cultures from *n* = 8 FBTEC donors. The resistance is presented as Ohms (Ω)/cm^2^. (**D**) Club cell quantification. Immunofluorescent staining of club cells (SCGB1A1, red) in ALI day 28 cultures from *n* = 8 FBTEC donors. Nuclei are stained blue with DAPI. Scale bar = 50 µm. (**E**) Ciliated cell quantification. Immunofluorescent staining of ciliated cells (acetylated tubulin, green) in ALI day 28 cultures from *n* = 8 FBTEC donors. Nuclei are stained blue with DAPI. Scale bar = 50 µm. Representative images from *n* = 3 FBTEC donors (1–3) are shown for Panels (**D**,**E**). (**F**) Box plots showing expression (on a log scale) of specific markers for BC (KRT5 and TP63), intermediate (KRT4 and KRT8), club (SCGB1A1), goblet (MUC5B) and ciliated (MYB and DNAH11) cells in ALI day 28 cultures from *n* = 8 FBTEC donors by bulk RNA-Seq. Each donor is represented by an asterix (*), with the bar and box representing the median expression and interquartile range (IQR) across all donors, and with whiskers extending to 1.5× the IQR in either direction from the top or bottom quartile.

To characterize the stem/progenitor capacity of the in vitro expanded FBTECs, we utilized the ALI culture model to study the differentiation of FBTECs into a mucociliary airway epithelium (Figure 2A). Histological analysis of ALI day 28 cultures demonstrated the presence of a multi-layered epithelium with BCs (KRT5+) and ciliated (acetylated tubulin+) cells (Figure 2B). The ability of the FBTEC cultures to establish tight junctions and maintain barrier integrity was confirmed via measurement of TEER (Figure 2C). Quantification of FBTEC differentiation into club (SCGB1A1+) and ciliated (acetylated tubulin+) cells demonstrated each cell type represented on average 20.9% (±3.7%) and 33.4% (±3.7%) of the total cell population, respectively (Figure 2D,E). Similar to our staining of in vivo fetal baboon trachea, we were unable to detect the presence of MUC5AC+ goblet cells in ALI day 28 cultures of FBTECs (data not shown). However, to further support our histological analysis, we performed bulk RNA-Seq of each FBTEC donor at ALI day 28 to analyze the expression of cell-type specific markers at the mRNA level. In addition to markers for BC (KRT5 and TP63), club (SCGB1A1) and ciliated (MYB and DNAH11) cells, we also detected the expression of markers for intermediate (KRT4 and KRT8) cells (Figure 2F). Unfortunately, we were unable to assess the expression of MUC5AC due to a failure to match this gene to the current sequence annotations. However, we detected expression of MUC5B, suggesting that ALI day 28 cultures of FBTECs may contain goblet cells (Figure 2F). In summary, our data demonstrate that FBTECs can be successfully isolated from in vivo tracheal epithelial samples, expanded in vitro and differentiated into a mucociliary airway epithelium on ALI culture.

### 3.2. ALI-Differentiated FBTECs Are Permissive to SARS-CoV-2 Infection

**Figure 3:**
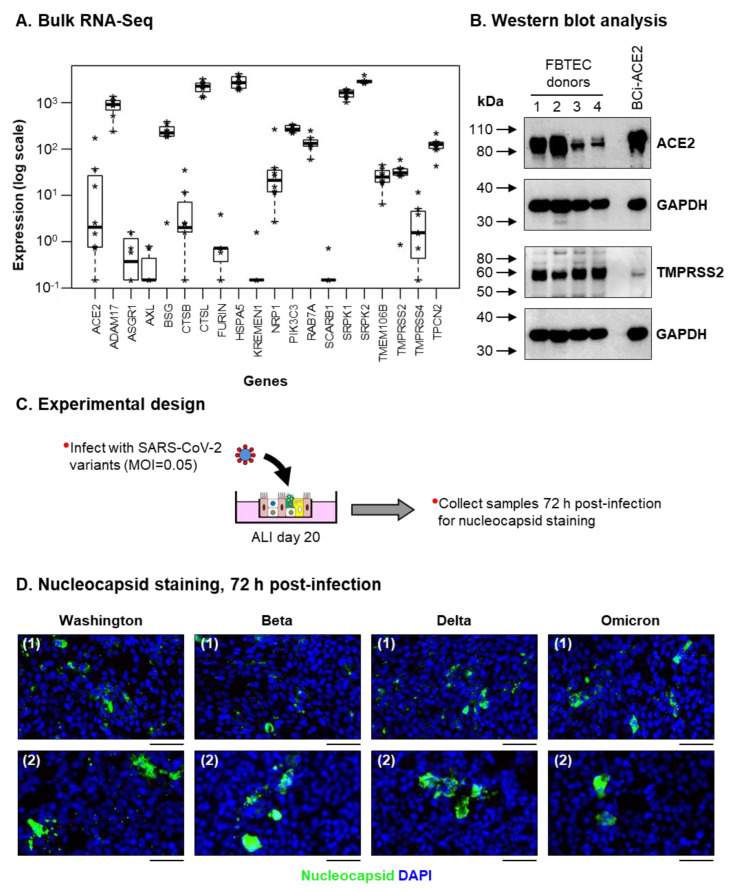
ALI differentiated FBTECs are permissive to SARS-CoV-2 Infection. (**A**) Box plots showing the expression (on a log scale) of host factors that regulate SARS-CoV-2 replication in ALI day 28 cultures from *n* = 8 FBTEC donors by bulk RNA-Seq. Each donor is represented by an asterix (*), with the bar and box representing the median expression and interquartile range (IQR) across all donors, and with whiskers extending to 1.5× the IQR in either direction from the top or bottom quartile. (**B**) Western blot analysis of ACE2 and TMPRSS2 protein levels in ALI day 28 differentiated cultures from *n* = 4 FBTEC donors (1–4). Cell lysates of the human airway epithelial cell line BCi-NS1.1 over-expressing human ACE2 (BCi-ACE2) were used as a positive control for both proteins. GAPDH was used as a loading control. (**C**) Experimental design. ALI day 20 cultures of FBTEC cells were infected with multiple SARS-CoV-2 variants (Washington, Beta, Delta and Omicron) at a MOI of 0.05. (**D**) Immunofluorescent staining of SARS-CoV-2 nucleocapsid (green) and the nuclei (blue, DAPI) in cultures 72 h post-infection for each SARS-CoV-2 variant (Washington, Beta, Delta and Omicron). Scale bar = 50 µm. Representative images from *n* = 2 FBTEC donors (1–2) are shown.

Infection of SARS-CoV-2 in the upper human respiratory tract is predominantly mediated by binding of the viral spike protein to the host receptor ACE2 located on the surface of ciliated cells [15,61]. Upon receptor binding, cellular proteases (including CTSL, FURIN and TMPRSS2) mediate proteolytic priming of the spike protein, which triggers fusion of the viral envelope with the cell membrane and subsequent release of the viral genome into the cytoplasm of the host cell, where it initiates its replication cycle [15,61]. To date, multiple host factors have been shown to regulate SARS-CoV-2 replication at multiple stages of the virus life cycle [15,61,62,63]. Bulk RNA-Seq analysis demonstrated ALI day 28 cultures of FBTEC cells expressed a large number of these factors, including ACE2, ADAM17, ASGR1, AXL, BSG, CTSB, CTSL, FURIN, HSPA5, NRP1, PIK3C3, RAB7A, SRPK1, SRPK2, TMEM106B, TMPRSS2, TMPRSS4 and TPCN2 (Figure 3A). However, we observed low expression for some factors (KREMEN1 and SCARB1) (Figure 3A). In support of the bulk RNA-Seq data, western blot analysis of ALI day 28 FBTEC cultures demonstrated expression of both ACE2 and TMPRSS2 (Figure 3B). Combined, these data suggest the cultures are potentially permissive to SARS-CoV-2 infection. To test this hypothesis, ALI cultures of FBTECs were infected apically with multiple SARS-CoV-2 variants (Washington, Beta, Delta and Omicron) at an equal MOI and harvested 72 h post-infection for staining of viral nucleocapsid protein (Figure 3C). Infection of differentiated ALI cultures generated from two independent FBTEC donors demonstrated positive staining for viral nucleocapsid 72 h post-infection with each variant (Figure 3D). While previous studies have identified differences in the fusogenic capacity of SARS-CoV-2 variants and their ability to induce cell fusion and syncytia formation in infected cells [47,64,65], we did not observe any gross differences in the pattern of nucleocapsid staining between the different variants following infection of FBTEC cells. Combined, these data suggest that ALI-differentiated FBTEC cultures express the SARS-CoV-2 host entry factors and are permissive to SARS-CoV-2 infection.

### 3.3. SARS-CoV-2 Infection of ALI-Differentiated FBTECs Induces a Proinflammatory Response

**Figure 4:**
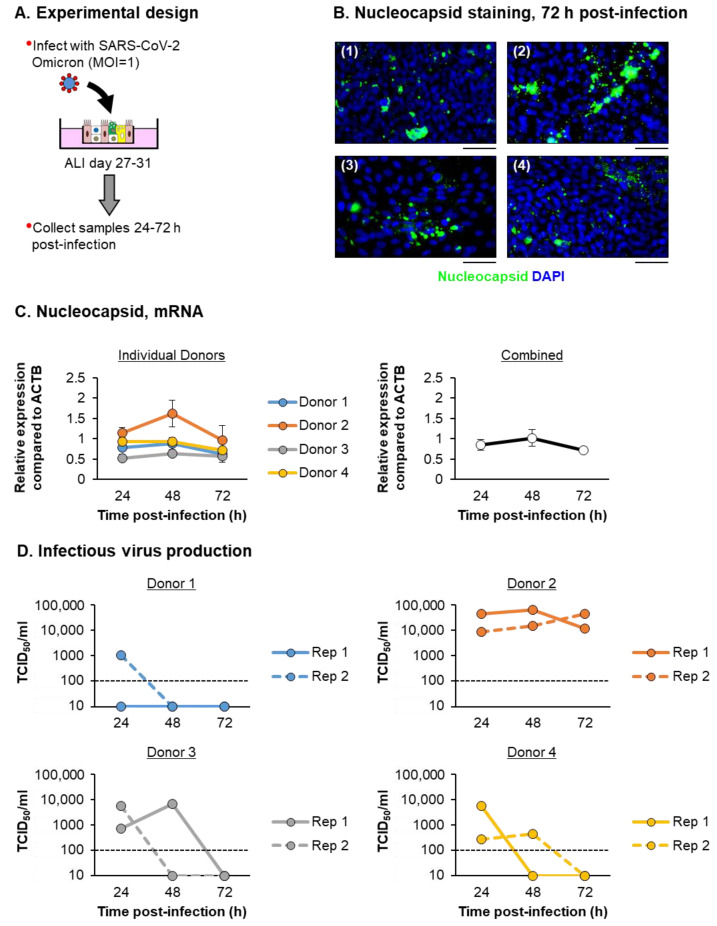
Temporal kinetics of SARS-CoV-2 Omicron infection. (**A**) Experimental design. ALI days 27–31 cultures of FBTEC cells from *n* = 4 donors (1–4) were infected with SARS-CoV-2 Omicron at a MOI of 1. The cells were then harvested as a function of time post-infection (24–72 h) for an analysis of the viral replication. (**B**) Immunofluorescent staining of SARS-CoV-2 nucleocapsid (green) and the nuclei (blue, DAPI) in cultures 72 h post-infection for each donor (1–4). Scale bar = 50 µm. (**C**) qPCR analysis of the SARS-CoV-2 nucleocapsid gene expression. In the left panel, the data are presented for each individual donor, with each data point representing the mean relative expression from *n* = 3 ALI wells and the error bars indicating the SEM. In the right panel, the data are combined and presented as the mean relative expression from *n* = 4 donors, with the error bars indicating the SEM. (**D**) Infectious virus production quantified by TCID_50_. Data are presented for each individual donor from two replicates (Rep 1 and Rep 2), with each data point representing the TCID_50_/mL calculated from a single ALI well. The black dashed line represents the limit of detection for the assay.

Since its identification in 2021, the Omicron variant has become the predominant circulating strain of SARS-CoV-2 worldwide [66]. Therefore, we used this variant to further characterize the virus-host interactions of SARS-CoV-2 with ALI-differentiated FBTEC cultures from *n* = 4 independent donors. To minimize experimental variability, the FBTEC cultures from all four donors were infected at the same time with the same stock of virus and harvested at the appropriate time points in tandem. To this end, ALI cultures were infected apically with SARS-CoV-2 Omicron at a MOI of 1 and harvested as a function of time (24–72 h) post-infection (Figure 4A). The airway epithelium derived from each FBTEC donor was readily infected with SARS-CoV-2, as indicated by staining for the viral nucleocapsid protein in the cultures 72 h post-infection (Figure 4B). Expression of the virus nucleocapsid gene was quantified as a function of time post-infection as a surrogate marker for virus replication (Figure 4C). While differences in the magnitude of the expression were observed between FBTEC donors, there appeared to be a consistent trend of increasing nucleocapsid expression from 24–48 h post-infection, with peak levels reached at 48 h, followed by a decrease between 48–72 h. To further characterize virus replication, we next quantified the infectious virus production from the apical chamber of two independent ALI wells at the same time points. In contrast to the nucleocapsid gene expression, we observed a high degree of variability in the production of infectious virus between each FBTEC donor (Figure 4D). Infectious virus production was the highest for donor 2, with infectious virus detected at each time point in both replicates and peak virus load observed at 48 h post-infection. However, while we were able to detect infectious virus in both replicates for donors 3 and 4 at 24 h post-infection, at 48 h we began to observe variability between the replicates and by 72 h post-infection, the virus load was below the limits of detection for both donors. Finally, while infectious virus was detected in one replicate at 24 h post-infection for donor 1, no infectious virus was detected at any other time point post-infection.

**Figure 5:**
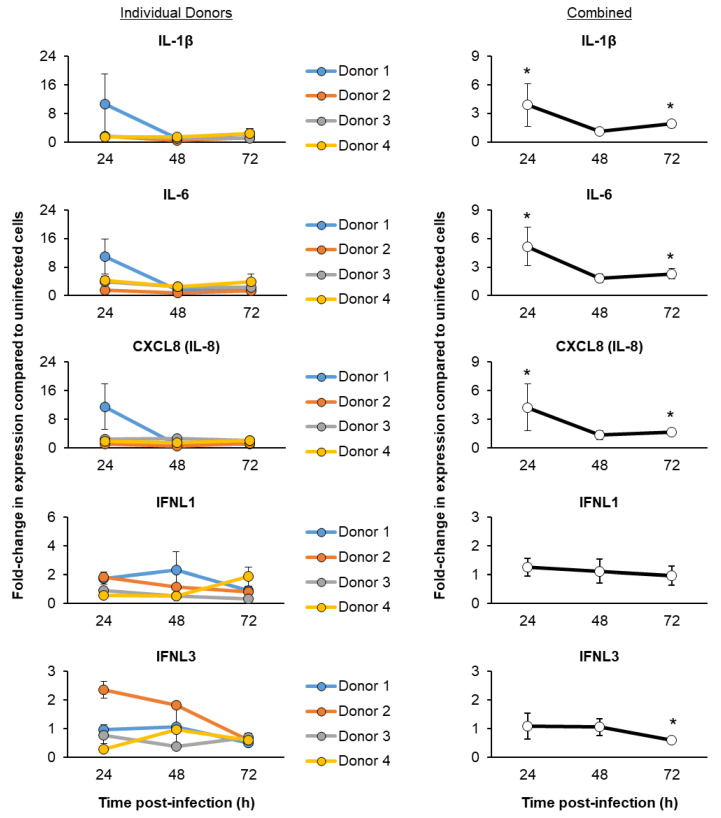
Temporal kinetics of the host cytokine and chemokine responses following SARS-CoV-2 Omicron infection. ALI day 27–31 cultures of FBTEC cells from *n* = 4 donors (1–4) were infected with SARS-CoV-2 Omicron at a MOI of 1. The cells were then as a function of time post-infection (24–72 h) for qPCR analysis of the immune related genes IL-1β, IL-6, CXCL8 (IL-8), IFNL1 and IFNL3. In the left panel, the data are presented for each individual donor, with each data point representing the mean fold-change in expression compared to uninfected cells from *n* = 3 ALI wells and the error bars indicating the SEM. In the right panel, the data are combined and is presented as the mean fold-change in expression compared to uninfected cells from *n* = 4 donors, with the error bars indicating the SEM. * *p* < 0.05.

In response to virus infection, airway epithelial cells produce a number of proinflammatory cytokines, chemokines and IFN-associated mediators to facilitate the recruitment of immune cells from the periphery of the lung and directly suppress viral replication via cell-intrinsic and cell-extrinsic mechanisms [10,11,12,13,14,15]. To evaluate the innate immune response of ALI-differentiated FBTECs to SARS-CoV-2 Omicron infection, we next performed targeted qPCR analysis of cytokines, chemokines and IFN signaling-associated genes. Specific genes were chosen based on the availability of primers for the detection of baboon genes and their previous association with SARS-CoV-2 infection and COVID-19 (Figure 5) [12,15,67,68,69].

Similar to studies with primary human airway epithelial cells, variations in the magnitude and kinetics of induction of the immune response were observed for each FBTEC donor following SARS-CoV-2 infection [49,55,70,71,72]. This was especially evident at the 24 h post-infection. For example, compared to donors 2–4, donor 1 displayed a higher level of induction of multiple cytokine and chemokine genes (IL-1β, IL-6 and CXCL8) (Figure 5). Similarly, compared to donors 1, 3 and 4, donor 2 displayed a higher level of IFNL3 induction (Figure 5). Despite the inter-donor differences, when combining the data for all four donors, we observed a consistent trend of induction for each immune mediator, which demonstrated that SARS-CoV-2 infection of ALI-differentiated cultures leads to early induction of proinflammatory cytokines and chemokines (IL-1β, IL-6 and CXCL8), but minimal induction of IFNs (IFNL2 and IFNL3).

**Figure 6:**
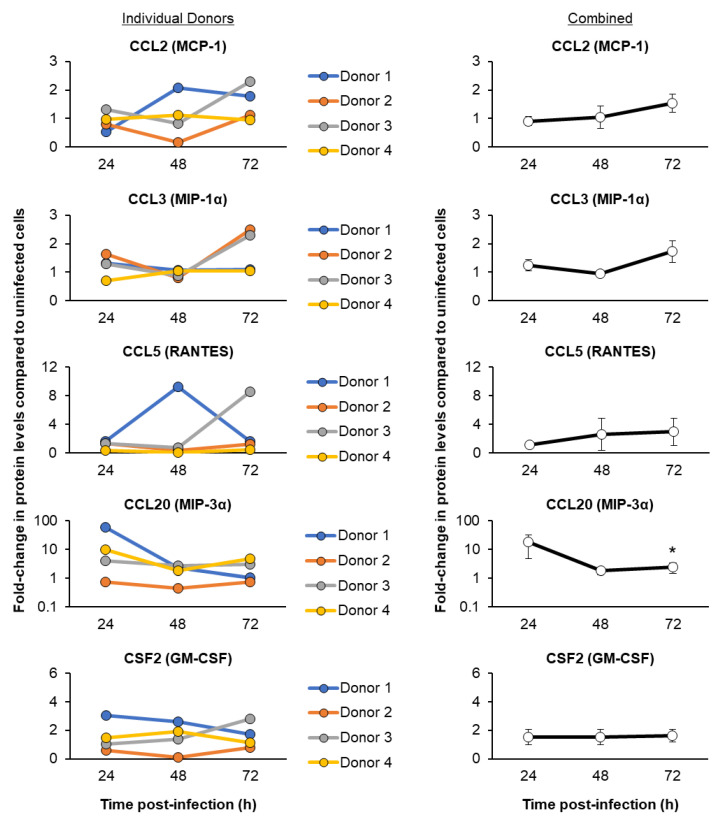
Temporal kinetics of the host cytokine and chemokine responses following SARS-CoV-2 Omicron infection. On ALI days 27–31, cultures of FBTEC cells from *n* = 4 donors (1–4) were infected with SARS-CoV-2 Omicron at a MOI of 1. The media supernatants were then harvested as a function of time post-infection (24–72 h) to analyze the protein levels of immune related genes CCL2 (MCP-1), CCL3 (MIP-1α), CCL5 (RANTES), CCL20 (MIP-3α) and CSF2 (GM-CSF). In the left panel, the data are presented for each individual donor, with each data point representing the fold-change in protein levels compared to uninfected cells from a single (*n* = 1) ALI well. In the right panel, the data are combined and presented as the mean fold-change in protein levels compared to the uninfected cells from *n* = 4 donors. The error bars indicate the SEM. * *p* ≤ 0.05.

**Figure 7:**
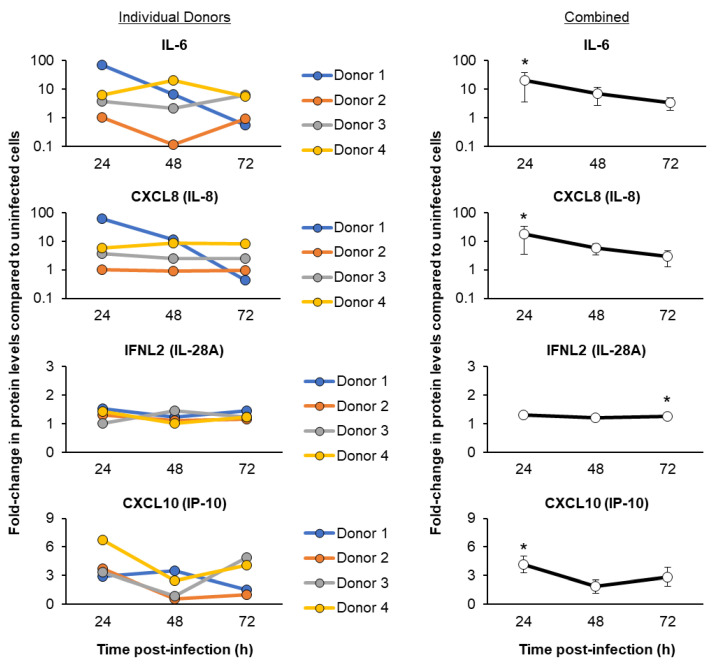
Temporal kinetics of the host cytokine, chemokine and IFN responses following SARS-CoV-2 Omicron infection. On ALI days 27–31, cultures of FBTEC cells from *n* = 4 donors (1–4) were infected with SARS-CoV-2 Omicron at a MOI of 1. The media supernatants were then harvested as a function of time post-infection (24–72 h) to analyze the protein levels of immune related genes IL-6, CXCL8 (IL-8), IFNL2 (IL-28A) and CXCL10 (IP-10). In the left panel, the data are presented for each individual donor, with each data point representing the fold-change in protein levels compared to uninfected cells from a single (*n* = 1) ALI well. In the right panel, the data are combined and presented as the mean fold-change in protein levels compared to the uninfected cells from *n* = 4 donors. The error bars indicate the SEM. * *p* ≤ 0.05.

To further characterize the host response to SARS-CoV-2 infection, we next quantified the protein levels of multiple cytokines, chemokines and IFN signaling-associated genes in the basolateral chamber culture media supernatants obtained from SARS-CoV-2-infected and uninfected cells (Figure 6 and Figure 7). Specific genes were chosen based on the availability of assays for the detection of baboon proteins and their previous association with SARS-CoV-2 infection and COVID-19 [12,15,67,68,69]. Similar to our qPCR analysis, we observed variations in the magnitude of induction of the immune response between individual FBTEC donors. However, when combining the data for all four donors, we observed a consistent trend of induction for each immune mediator. This included proteins (CCL20, IL-6, CXCL8 and CXCL10) with peak-levels of induction early (24 h) post-infection, proteins (CCL2, CCL3 and CCL5) with a delayed response that show peak levels of induction late (72 h) post-infection, and proteins that display minimal to no induction (CSF2 and IFNL2) throughout infection (24–72 h) (Figure 6 and Figure 7). Importantly, the induction kinetics of IL-6 and CXCL8 post-infection are consistent at the mRNA and protein levels in our study (Figure 5 and Figure 7), which further strengthens the robustness of our data.

In summary, these findings indicate that the SARS-CoV-2 Omicron infection of differentiated FBTEC cultures leads to a potent host response, including the early induction of multiple cytokines and chemokines, and minimal induction of IFN signaling.

## 4. Discussion

The use of NHP models in the study of human respiratory virus infection, including SARS-CoV-2, has proved critical to furthering our knowledge of the mechanisms that regulate virus pathogenesis and played a significant role in the development of therapeutics and vaccines to treat human disease [35,36,37,38,39,40,41,42,43]. However, despite their high degree of relatedness to human anatomy, physiology and immunology, NHPs can fail to fully recapitulate the human disease or may not be permissive to infection with the human virus [26,37,39,40,41,44]. Taking this into consideration, along with the important ethical considerations for the use of NHPs in biomedical research, the substantial cost of performing in vivo NHP studies and the current shortage of NHPs available for biomedical research in the United States [73], having an in vitro model of the mucociliary airway epithelium to characterize human respiratory virus infection in NHPs would be highly desirable and allow for the rapid characterization of virus tropism, virus replication and the host response. Furthermore, results from these in vitro studies can help determine the suitability of a specific NHP before proceeding with in vivo studies. Therefore, using the olive baboon as our NHP model, we have developed the methodology to isolate primary FBTECs from the trachea of baboon fetuses delivered by Cesarean section. Primary FBTECs can be expanded in vitro, cryopreserved and importantly, differentiated into a mucociliary airway epithelium reminiscent of that observed in vivo. As proof-of-principle to highlight the utility of this model, we demonstrate that differentiated FBTEC cultures are permissive to infection with SARS-CoV-2, resulting in the production of infectious virus and a potent host response.

Similar to previous in vitro ALI studies with primary human airway epithelial cells [49,55,70,71,72], we observed a high degree of variability in the kinetics and magnitude of SARS-CoV-2 virus replication and induction of the immune response between different FBTEC donors. Furthermore, for three of the FBTEC donors, we failed to detect the production of infectious virus at 72 h post-infection, despite detecting the expression of the virus nucleocapsid. To minimize experimental variability, the FBTEC cultures from all four donors were infected at the same time with the same stock of virus. Therefore, this variability likely results from inherent biological differences between the cultures (i.e., expression levels of the host receptor/entry components and/or cell intrinsic genetic factors specific to each donor), which is not surprising considering the outbred nature of the donor NHPs. These inherent biological differences may impact multiple stages of the virus life cycle, including entry, replication, release, spread and subsequently the host cells innate immune response to virus infection [12,15,61]. In support of this, our bulk RNA-Seq analysis of the ALI day 28 FBTEC cultures identified donor-specific differences in the expression levels of multiple host factors that regulate SARS-CoV-2 replication in human cells (i.e., ACE2, CTSB and TMPRSS4). Low expression levels of certain factors may impair the efficiency of virus replication and spread, which in turn may limit the magnitude of virus propagation at late time points (i.e., 72 h) post-infection due to suppression of virus replication by the host’s innate immune response. Despite the inter-donor differences, when combining the data from all the FBTEC donors, we observed a consistent trend of induction of proinflammatory cytokines (i.e., CCL20 and IL-6), chemokines (i.e., CCL5, CXCL8 and CXCL10) previously associated with SARS-CoV-2 infection (in vitro and in vivo), and the COVID-19-associated “cytokine storm” in humans [12,15,67,68,69]. Consistent with SARS-CoV-2 infection of human airway epithelial cells, we observe a rapid proinflammatory response during the early stages (24 h) of infection characterized by an increased expression of multiple cytokines and chemokines (e.g., CCL20, IL-6, CXCL8 and CXCL10). Despite the limited analysis of IFN signaling components (IFNL1, 2 and 3), we observed no induction of the type III IFN response in SARS-CoV-2-infected FBTECs, which contrasts with the findings of a delayed IFN response previously reported in SARS-CoV-2 infection of primary human airway epithelial cells by our group and others [55,74,75]. While these contrasting findings may be related to species specific differences between the studies (i.e., baboon vs. human), the use of fetal vs. adult cells, or SARS-CoV-2 virus strains (i.e., Washington vs. Omicron), this discrepancy in IFN induction may also reflect technical differences between each study (i.e., low vs. high virus MOI used) or the lack of type I IFN analysis in our study.

Baboons have been successfully used as an in vivo NHP model for multiple human infectious diseases, including Bordetella pertussis, Zika virus, RSV and more recently SARS-CoV-2 [41,76,77,78,79,80,81,82]. A recent study by Singh et al., [41] compared the responses of rhesus macaques, marmosets and baboons to acute infection with SARS-CoV-2 and demonstrated that baboons are susceptible to SARS-CoV-2 and develop more extensive pulmonary pathology associated with the human disease compared to rhesus macaques and marmosets. In addition, the authors demonstrated that SARS-CoV-2 infection leads to the induction of cytokines, chemokines, and IFN-associated proteins relevant to COVID-19 in humans. Therefore, our in vitro study supports these findings and further validates the use of baboons as a physiologically relevant NHP model to study SARS-CoV-2.

In summary, our study demonstrates the feasibility of isolating primary FBTECs for subsequent in vitro expansion, cryopreservation, and differentiation on ALI cultures. Furthermore, we demonstrate that in vitro ALI-differentiated FBTECs cultures are permissive to SARS-CoV-2 infection and lead to the production of infectious virus and a potent host innate immune response. However, we acknowledge there are some limitations to our study. First, compared to our prior in vitro study modeling SARS-CoV-2 infection in ALI cultures of human bronchial epithelial cells [55], infection of FBTECs resulted in limited virus spread and reduced virus production at later time points. Therefore, it’s possible that FBTECs may not fully replicate the SARS-CoV-2 replication cycle in human cells due to lack of expression of certain host factors required for efficient SARS-CoV-2 replication and/or that the baboon homologues of these proteins have differing cellular functions. Second, our study lacks cells from adult baboons, which prevented us from comparing the stem/progenitor capacity and SARS-CoV-2 virus-host interactions of fetal vs. adult baboons. Human adults are more susceptible than children to SARS-CoV-2 infection and the development of severe disease [83,84]. In addition, a comparison of proinflammatory cytokines, chemokines and IFN-associated protein levels in the serum and bronchoalveolar lavage of old vs. young baboons infected with SARS-CoV-2 demonstrated a higher magnitude of induction in the older animals [41]. Therefore, the use of adult baboon tracheal airway epithelial cells may be better suited to studying the innate immune response to SARS-CoV-2 infection than fetal derived cells. Third, based on the limited availability of assays suitable for the detection of baboon genes and proteins, our analysis of the innate immune response of FBTECs to SARS-CoV-2 infection was limited to a small number of genes previously associated with SARS-CoV-2 infection and COVID-19 [12,15,67,68,69] and lacked those related to type I IFN signaling. Finally, we acknowledge there are important ethical and cost considerations associated with obtaining fetal tissue from pregnant baboons. To reduce these factors, the study described in this manuscript was added onto an existing NHP study to maximize the use of the fetal tissue available. Now that we have successfully isolated and cryopreserved primary FBTECs, our long-term goal is to establish immortalized cell lines from the donors we have already isolated. This would eliminate the need to collect cells from additional fetuses and reduce the ethical concerns associated with this research. Furthermore, it would provide an important research tool that could be disseminated to the wider research community. Despite these limitations, we have successfully developed an in vitro NHP model of the mucociliary airway epithelium using primary FBTECs that provides a platform for the future study of virus-host interactions in the context of SARS-CoV-2 infection and potentially other human respiratory viruses, including IAV, rhinovirus and RSV.

## Data Availability

The raw data from the bulk RNA-Seq studies are publicly available at the Gene Expression Omnibus (GEO) site (http://www.ncbi.nlm.nih.gov/geo/, accessed on 7 March 2023), accession number GSE226820.

## Supplementary Materials

The following supporting information can be downloaded at: https://www.mdpi.com/article/10.3390/v15040862/s1, Supplementary Figure S1. Original western blot images of ACE2 and GAPDH protein levels in *n* = 4 FBTEC donors (1–4) differentiated on ALI culture. At ALI day 28, the cells were harvested for analysis. Cell lysates of the human airway epithelial cell line BCi-NS1.1 over-expressing human ACE2 (BCi-ACE2) were used as a positive control. (A) ACE2 western blot images. (B) GAPDH western blot images. Supplementary Figure S2. Original western blot images of TMPRSS2 and GAPDH protein levels in *n* = 4 FBTEC donors (1–4) differentiated on ALI culture. At ALI day 28, the cells were harvested for analysis. Cell lysates of the human airway epithelial cell line BCi-NS1.1 over-expressing human ACE2 (BCi-ACE2) were used as a positive control. (A) TMPRSS2 western blot images. (B) GAPDH western blot images.
