# Supplementary material for: The Isolation and In Vitro Differentiation of Primary Fetal Baboon Tracheal Epithelial Cells for the Study of SARS-CoV-2 Host-Virus Interactions
Source: Viruses. 2023 Mar 28;15(4):862. doi: 10.3390/v15040862 (PMC10146425; doi:10.3390/v15040862)
Supplement: Supplementary file 1 [file viruses-15-00862-s001.zip › viruses-2065240-supplementary.pdf]

A. ACE2 western blot images

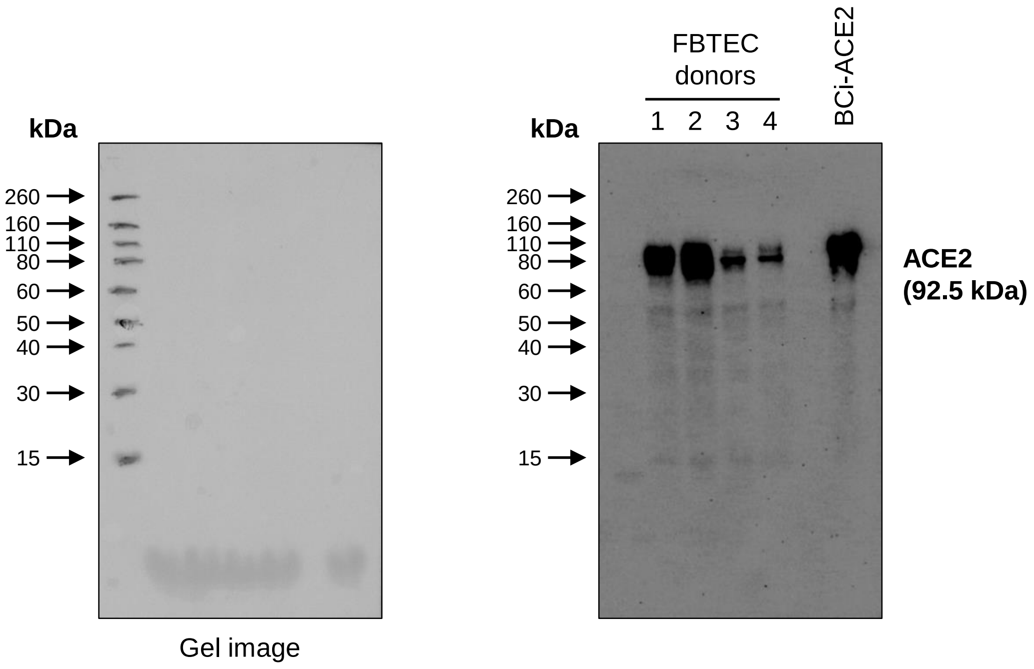

B. GAPDH western blot images

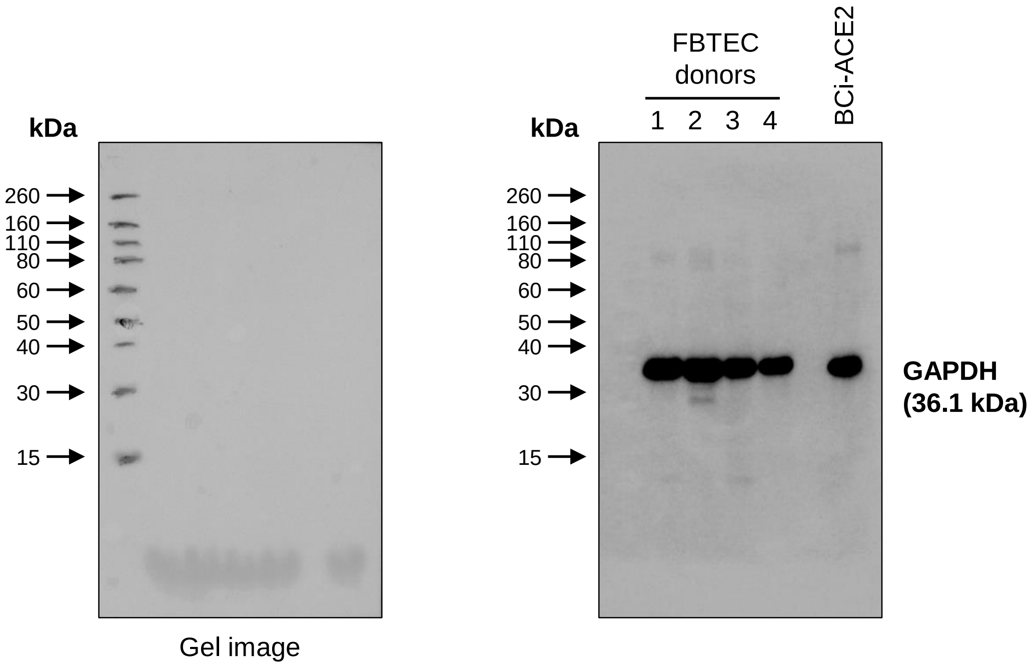

Supplementary Figure S1. Original western blot images of ACE2 and GAPDH protein levels in n = 4 FBTEC donors (1-4) differentiated on ALI culture. At ALI day 28, the cells were harvested for analysis. Cell lysates of the human airway epithelial cell line BCi-NS1.1 over-expressing human ACE2 (BCi-ACE2) were used as a positive control. (**A**) ACE2 western blot images. (**B**) GAPDH western blot images.

Supplementary Figure S2

A. TMPRSS2 western blot images

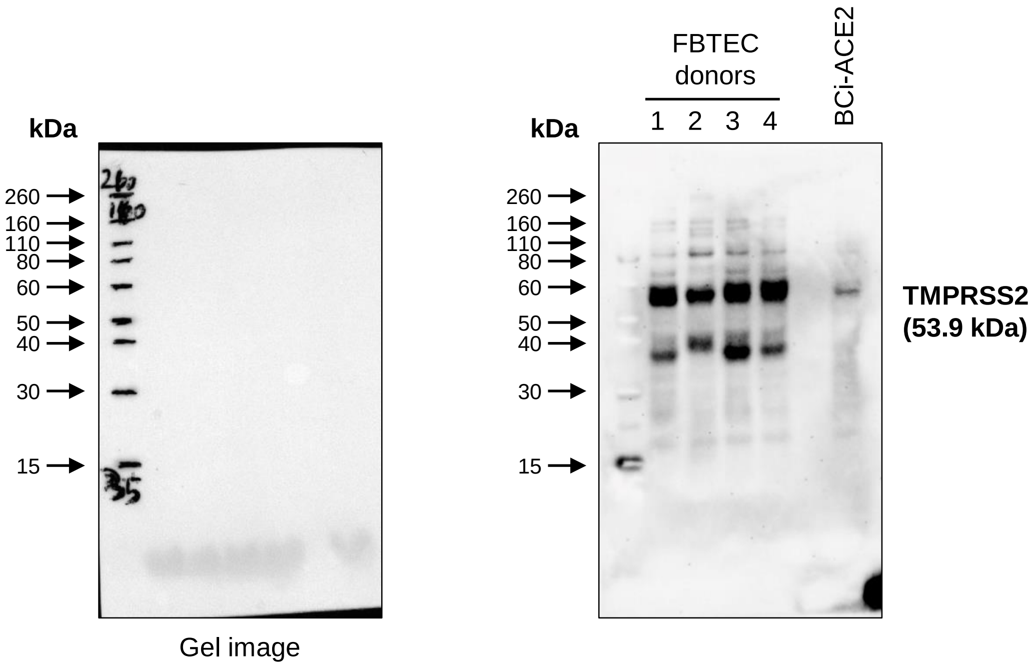

B. GAPDH western blot images

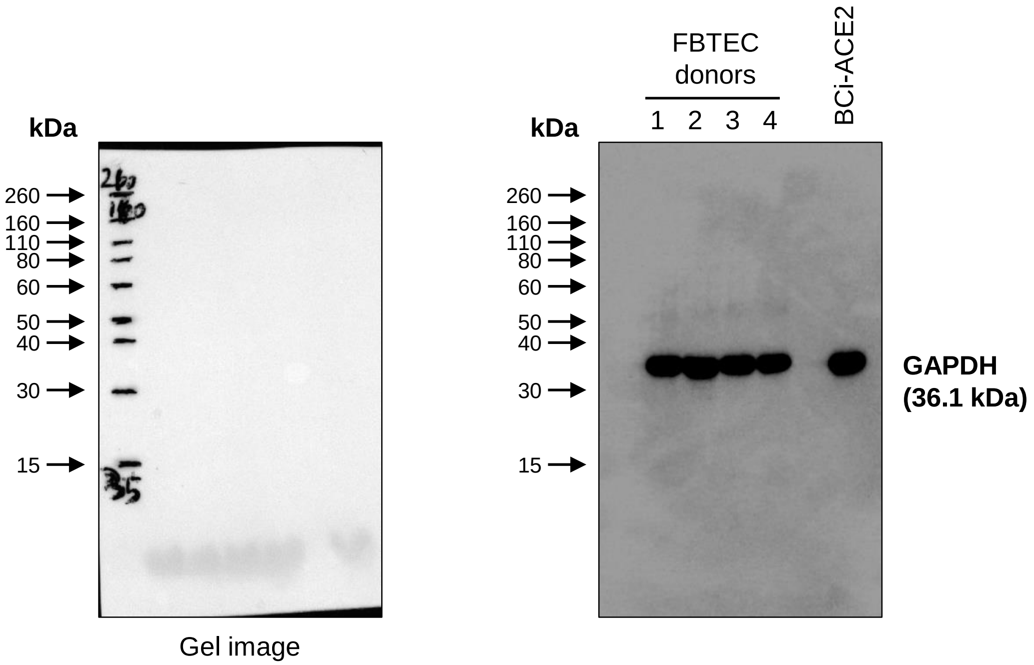

Supplementary Figure S2. Original western blot images of TMPRSS2 and GAPDH protein levels in n = 4 FBTEC donors (1-4) differentiated on ALI culture. At ALI day 28, the cells were harvested for analysis. Cell lysates of the human airway epithelial cell line BCi-NS1.1 over-expressing human ACE2 (BCi-ACE2) were used as a positive control. (**A**) TMPRSS2 western blot images. (**B**) GAPDH western blot images.
